# Nuanced Considerations in Immediate Versus Staged Multivessel PCI: Implications for Clinical Practice and Guideline Development

**DOI:** 10.1002/clc.70224

**Published:** 2025-11-26

**Authors:** Hassan E Muhammad, Samia Nawaz

**Affiliations:** ^1^ Gujranwala Medical College Gujranwala Pakistan; ^2^ University College of Medicine and Dentistry Lahore Pakistan

To the Editor,

The recent systematic review and meta‐analysis by Yasmin et al. addressing immediate versus staged revascularization of non‐culprit arteries in patients with acute coronary syndrome (ACS) and multivessel disease offers timely and clinically relevant insights [1]. While their work advances the field, several important considerations remain under explored and warrant further attention.

Procedural complexity is an underappreciated determinant of outcomes. Immediate multivessel intervention often entails longer procedural duration, higher fluoroscopy exposure, and greater operator workload factors rarely captured in randomized trials. These elements are directly linked to complication rates and periprocedural safety. Contemporary evidence demonstrates that operator experience and intravascular imaging improve long‐term outcomes in complex PCI, underscoring the importance of workload and expertise as modifiers of risk [2].

Renal outcomes also deserve greater emphasis. Although the authors note the potential for contrast‐induced nephropathy, their analysis does not account for cumulative contrast burden or the possibility of renal recovery between staged procedures. Observational studies have established that contrast volume is a strong predictor of acute kidney injury and adverse prognosis, particularly in patients with impaired baseline renal function [3, 4]. Pooled analyses that omit this dimension may underestimate renal risk associated with either strategy.

Economic and resource implications are highly relevant but absent from the analysis. Differences in hospital length of stay, catheterization laboratory occupancy, and downstream costs carry meaningful consequences for patients and health systems. Prior studies indicate that staged PCI is associated with higher cumulative costs compared with one‐time complete revascularization [5]. These considerations are particularly salient for resource‐limited settings where system efficiency influences real‐world adoption.

Physiologic lesion assessment is another key omission. The landmark FAME trial demonstrated that fractional flow reserve (FFR)‐guided PCI reduces unnecessary stenting and improves outcomes [6]. Current European guidelines endorse physiology‐guided revascularization whenever feasible [7]. Staged procedures allow for reassessment with FFR or instantaneous wave‐free ratio following stabilization, which may enhance the appropriateness of intervention and reduce overtreatment.

Finally, operator and institutional expertise are well‐recognized determinants of PCI outcomes. Large registries confirm that both operator volume and the use of intravascular imaging independently improve results [2]. Failure to stratify outcomes by these variables limits generalizability across diverse practice environments.

In conclusion, while the work by Yasmin et al. contributes significantly to the debate on immediate versus staged multivessel PCI, future analyses would benefit from integrating procedural complexity, renal endpoints, economic factors, physiologic lesion reassessment, and operator expertise.

**Prospective studies should** (a) quantify procedural duration, radiation, and operator workload; (b) systematically evaluate cumulative contrast exposure and recovery; (c) incorporate lesion‐specific physiologic assessment after stabilization; (d) assess health‐system and economic outcomes; and (e) stratify results by operator and institutional experience. Such efforts will strengthen the evidence base, refine guideline recommendations, and ensure that revascularization strategies remain safe, efficient, and adaptable across healthcare systems.

## Ethics Statement

ChatGPT‐4o (OpenAI) was used only for grammar and language refinement. All content was written and verified by the authors in accordance with the TITAN 2025 guideline.

## Conflicts of Interest

The authors declare no conflicts of interest.

## Data Availability

Data sharing not applicable to this article as no datasets were generated or analysed during the current study.
